# Characterization of a novel reassortant H5N6 highly pathogenic avian influenza virus clade 2.3.4.4 in Korea, 2017

**DOI:** 10.1038/s41426-018-0104-3

**Published:** 2018-06-13

**Authors:** Eun-Kyoung Lee, Yu-Na Lee, Soo-Jeong Kye, Nicola S. Lewis, Ian H. Brown, Mingeun Sagong, Gyeong-Beom Heo, Yong-Myung Kang, Hyun-Kyu Cho, Hyun-mi Kang, Sun-Ha Cheon, Myeongheon Lee, Bong-Kyun Park, Yong-Joo Kim, Youn-Jeong Lee

**Affiliations:** 1 Avian Influenza Research and Diagnostic Division, Animal and Plant Quarantine Agency, 177, Hyeoksin 8-ro, Gimcheon-si, Gyeongsangbuk-do 39660 Republic of Korea; 2 EU/OIE/FAO international reference laboratory for avian influenza, Animal and Plant Health Agency-Weybridge, Addlestone, Surrey KT15 3NB United Kingdom; 3 Department of Zoology, University of Cambridge, Downing Street, Cambridge, CB2 #EJ United Kingdom

Dear Editor,

Highly pathogenic avian influenza (HPAI) viruses of the H5 subtype remain a serious concern for both poultry and human health. The clade 2.3.4.4 H5 viruses have evolved into four genetic groups (nominally designated A, B, C, and D). Clade 2.3.4.4.A and B H5N8 viruses appear to have been reintroduced from poultry into wild bird populations, likely in Asia, and then spread globally through wild bird migration during 2014–2015 (2.3.4.4.A) and 2016–2017 (2.3.4.4.B)^1, 2^.

Clade 2.3.4.4.C and D H5 HPAI viruses have circulated exclusively in China and a few other countries in Southeast Asia. Indeed, clade 2.3.4.4.C H5N6 HPAI viruses have been the predominant clade detected in poultry populations in China^3^. Recently, clade 2.3.4.4 H5N6 subtypes have also been associated with human infection in China (http://www.wpro.who.int/emerging_diseases/AvianInfluenza/en/).

The clade 2.3.4.4.A H5N8 viruses were identified in China in 2013 and were subsequently introduced into Korea in early 2014^4, 5^. Since then, the spread of clade 2.3.4.4.A viruses mediated by wild bird migration has resulted in intra- and intercontinental transmission. These viruses then reassorted with low pathogenic avian influenza (LPAI) of the Eurasian or North American lineage to result in novel H5Nx subtypes (such as N1, N2, N3, N5, and N8), which subsequently spread broadly throughout Eurasia and North America^6, 7^.

In mid-2016, a novel clade of 2.3.4.4.B H5N8 viruses was detected in wild birds in Uvs-Nuur Lake in Siberia, Russia, near the border with Mongolia and in Qinghai Lake in China. These H5N8 viruses were novel reassortants consisting of five segments (polymerase basic-2 (PB2), polymerase basic-1 (PB1), polymerase acidic (PA), nucleoprotein (NP), and matrix (MP)) from Eurasian LPAI viruses most similar to H5N8 viruses isolated in China in 2013^8, 9^. These Russia–Mongolia H5N8 viruses then further reassorted with the NA genes from Eurasian LPAI viruses to result in additional H5N5 and H5N6 subtypes. This 2.3.4.4.B lineage has been detected in most European countries, Central Asia, the Middle East, and Africa during 2016–2017^10^.

In Korea, there have been four outbreaks of H5N1 HPAI from 2003 to 2011. However, since 2014, large epizootics in poultry have been caused by clade 2.3.4.4.A H5N8 viruses. Then, in late 2016, outbreaks in poultry resulted from clade 2.3.4.4.C H5N6 and clade 2.3.4.4.B H5N8 HPAI viruses, demonstrating the expanding diversity of the threat to the poultry industry in Korea^11, 12^.

In November 2017, we detected two novel H5N6 HPAI viruses, A/duck/Korea/HD1/2017(H5N6) (HD1) and A/mallard/Korea/Jeju-H24/2017(H5N6) (Jeju-H24), through our active surveillance program for avian influenza. On November 17, an H5N6 HPAI virus was detected in a broiler duck farm in Gochang of Jeonbuk Province (nominated HD1). The second H5N6 virus was isolated from fecal samples taken from a wild mallard (*Anas platyrhynchos*) collected in Jeju on November 27 (nominated Jeju-H24). Virus isolation, sequencing, and phylogenetic analysis were performed as described (Supplementary Materials and Methods 1). The two isolates shared 99.69–100% nucleotide homology across all eight genes. A BLAST search (http://www.ncbi.nlm.nih.gov/genomes/FLU/FLU.html) revealed that these novel H5N6 viruses were reassortants with H5 genes most genetically similar to clade 2.3.4.4.B viruses derived from European H5N8 HPAI viruses circulating during the 2016–2017 epizootic and N6 genes of a Eurasian LPAI virus lineage circulating in wild birds (Fig. 1, Supplementary Figure S1 and Table S1).

**Fig. 1:**
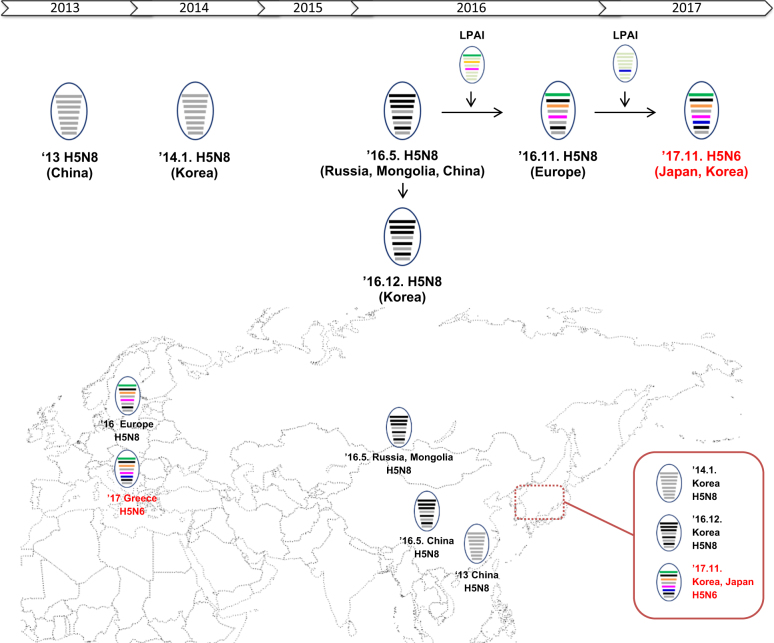
Evolutionary timeline of the genetic cassette of the current Korean 2.3.4.4B H5Nx highly pathogenic avian influenza viruses, generated through reassortment. Current reassortant H5N6 viruses consist of PB2, PB1, PA, HA, NP, M, and NS segments from European clade 2.3.4.4.B H5N8 viruses isolated in 2016 and N6 gene segments from low pathogenic avian influenza viruses. Gray colors represent the lineage of the segments originating from viruses of clade 2.3.4.4.B in 2013–2014, and the other colors denote the segments derived from low-pathogenic avian influenza virus through reassortment events. The eight horizontal bars indicate the gene segments in the order (top to bottom of the virion) PB2, PB1, PA, HA, NP, NA, M, and NS. HA hemagglutinin, M matrix, NA neuraminidase, NP nucleoprotein, NS nonstructural, PA polymerase acidic, PB1 polymerase basic 1, PB2 polymerase basic 2

Phylogenetic analyses of all genes except for the NA gene showed that the closest genetic relatives to the two viruses isolated in Korea were isolated from both wild and domestic birds sampled in a number of European countries in 2016/17. Furthermore, both the NP and NA genes were most similar to the H5N6 reassortant virus isolated from a chicken in Greece during this epizootic (Supplementary Figure S1 and S2).

The H5N6 viruses had multiple polybasic amino acid motifs at the HA cleavage site, characteristic of highly pathogenic influenza viruses (PLRERRRKR/GLF). Interestingly, the HPAI cleavage site motif differed between the Greek and the Korean clade 2.3.4.4.B strains. However, it is identical to the Japanese H5N6 HPAI virus (A/mute swan/Shimane/3211A001/2017) isolated from a wild bird on 5 November 2017, perhaps indicating heterogeneity in the emerging 2.3.4.4.B H5N6 lineage that is potentially being maintained in wild birds.

The clade 2.3.4.4.B viruses have consistently evolved into novel H5Nx subtypes with various gene constellations by reassortment with LPAI viruses since 2013. In particular, the internal gene segments derived from Eurasian LPAI viruses in wild birds that have reassorted with the clade 2.3.4.4 ‘H5N8 backbone’ and the H5N5 and H5N8 HPAI subtypes of various genotypes were detected in Germany, Italy, the Netherlands, Croatia and the Republic of Georgia^13–15^. Reassortant H5Nx viruses might diffuse among wild bird species in Eurasian breeding or staging sites, such as Central Asia and Siberia, and these viruses then disseminate into Eastern Asia (Korea and Japan). Conversely, there might be some degree of temporo-spatial segregation across Eurasian wild bird populations, resulting in maintenance of distinct variants and, therefore, heterogeneity in emerging strains in different geographic regions. These hypotheses are, as yet, difficult to test owing to significant sampling biases in wild bird influenza monitoring and would require more strategically targeted surveillance in ecologically relevant populations.

The novel clade 2.3.4.4.B H5N6 viruses isolated in November 2017 were phylogenetically distinguishable from the clade 2.3.4.4 viruses (H5N6 and H5N8) previously isolated in Korea during 2016–2017. In late 2016, clade 2.3.4.4.C H5N6 HPAI viruses that originated from China were introduced to Korea and Japan by wild birds and caused enormous economic losses to the poultry industry, particularly in Korea^12^. Recent H5N6 viruses from 2017 showed <95% homologies in eight gene segments with previous Korean H5N6 viruses isolated in 2016. In addition, novel H5N6 viruses showed <97% in nucleotide identities with three genes (PB2, PA, and NP) with previous Korean H5N8 viruses of clade 2.3.4.4.B in the 2016/2017 winter season. These results indicate that new H5N6 viruses are unlikely to have been circulating undetected in Korea; therefore, they are more likely to represent a new introduction mediated by migratory birds.

In this study, we reported novel reassortant H5N6 HPAI viruses isolated from wild birds and poultry in November 2017. Genetic analysis indicated that these isolates had been generated through reassortment events between European H5N8 HPAI viruses in 2016/2017, which were descendants of Russia–Mongolia H5N8 viruses, and Eurasian lineage LPAI viruses. Novel H5N6 viruses with similar gene constellations were founded in both Greece and Korea, potentially derived by new reassortant events in Eurasian wild bird populations at breeding sites or staging sites and then spread to multiple geographic regions through different migratory subpopulations. More detailed information on HPAI viruses identified through active surveillance programs throughout Eurasia is needed to better evaluate the ecology, evolution, dynamics and diffusion of these viruses in wild birds, particularly in breeding sites in Mongolia and Siberia where these species likely converge in the summer with the potential for reassortment-driven expansion in diversity, and to assess the potential risks to animal and public health.

## Electronic supplementary material

Supplementary Materials and Methods

Supplementary Figure S1

Supplementary Figure S2

Supplementary table S1
