# Supplementary material for: Characterization of a novel reassortant H5N6 highly pathogenic avian influenza virus clade 2.3.4.4 in Korea, 2017
Source: Emerg Microbes Infect. 2018 Jun 13;7:103. doi: 10.1038/s41426-018-0104-3 (PMC5997646; doi:10.1038/s41426-018-0104-3)
Supplement: Supplementary file 1 — Supplementary Materials and Methods [file 41426_2018_104_MOESM1_ESM.doc]

**Supplementary Materials and Methods**

**Virus isolation and Sequencing**

Viral RNA extracted from organs (trachea, cecal tonsil and kidney) of dead birds or allantoic fluids using a Patho Gene-spin DNA/RNA extraction kit (iNtRON Biotechnology, Korea) by according to the manufacturer’s instructions were positive for H5 and N6 subtype by RT-PCR (1, 2). For virus isolation, the specimens were inoculated into embryonated specific pathogen-free chicken eggs. After incubation at 37℃, allantoic fluid from inoculated eggs was harvested and tested using the hemagglutination assay. All eight RNA genomic segments were amplified using segment specific primers and directly sequenced (3). The nucleotide sequences of the eight RNA genomic segments of two H5N6 isolates, A/duck/Korea/HD1/2017(H5N6) (HD1) and A/mallard/Korea/Jeju-H24/2017(H5N6) (Jeju-H24), were deposited to GISAID (global initiative on sharing all influenza data). (Accession numbers: EPI1123314-21 and EPI1123332-39). In addition, the nucleotide sequences of two H5N8 viruses (A/chicken/Gimje2/2017(H5N8) and A/chicken/Gunsan/2017(H5N8)), isolated in February and June 2017, were deposited to GISAID (EPI1123348-55 and EPI1123340-47), respectively. The Greek isolate, A/chicken/Greece/39_2017/2017(H5N6) was also deposited into GISAID (EPI1122874-881).

**Phylogenetic analyses**

The nucleotide sequences for phylogenetic analysis were downloaded from GISAID ([http://www.gisaid.org](http://www.gisaid.org/)) and from GenBank on 8 December 2017. We aligned sequences from each gene segment separately using MAFFT v7.305b (Katoh and Standley, 2013) and trimmed in Aliview v1.18. We inferred maximum likelihood (ML) phylogenetic trees for each gene segment using IQ-TREE, 1.5.5.5 (Nguyen et al., 2015) and ModelFinder (Kalyaanamoorthy et al., 2017) and obtained branch supports with SH-like approximate likelihood ratio test (aLRT) and standard non-parametric bootstrap with 1000 replications. All trees were visualised in FigTree v1.4.2, with mid-point rooting and increasing node-order. Phylogenetic trees constructed for each segment are shown in Supplementary Figure S1 and S2. In Supplementary Figure S1 (A), the representative viruses among clade 2.3.4.4 H5 viruses (subgroup A, B, C and D) were selected to focus dataset on contemporary strains in Asia. In Supplementary Figure S1 (B), N6 sequences including H5N6 subtypes and Eurasian HxN6 LPAI viruses were likewise included to infer phylogenetic relationships among contemporary strains. Conversely, Supplementary Figure S2 utilized an inclusive whole genome dataset with the closest similarity. The two datasets have some similarity but whole genomes were not available for all sequences in the focuses Asian dataset, and neither did some HxN6 LPAI viruses have whole genome data.

Supplementary Figure S1. **Maximum-likelihood Phylogenetic tree of the H5 and N6 gene segments for novel H5N6 HPAI.** (A) hemagglutinin (HA) and (B) neuraminidase (NA). The highly pathogenic and low pathogenic influenza virus sequences from the GISAID EpiFlu^TM^ database (http://platform.gisaid.org/epi3/frontend#326742) and Genbank (http://www.ncbi.nlm.nih.gov/genbank) were used for each phylogenetic comparison. The genetic subclades are annotated to the right of the tree. At each branch, the number indicates a bootstrap value. Black circle and Triangular index indicate the H5N6 HPAI isolates in Korea and the H5N6 HPAI virus in Greece, respectively. The scale bar indicates nucleotide substitutions per site.

Supplementary Figure S2**. Maximum-likelihood Phylogenetic tree for 8 gene segments for the novel H5N6 viruses with highly similar sequences.** (A) polymerase basic-2 (PB2), (B) polymerase basic-1 (PB1), (C) polymerase acidic (PA), (D) hemagglutinin, (E) nucleoprotein (NP), (F) neuraminidase, (G) matrix (MP), and (H) nonstructural (NS). The highly pathogenic and low pathogenic influenza virus sequences from the GISAID EpiFlu^TM^ database (http://platform.gisaid.org/epi3/frontend#326742) were used for each phylogenetic comparison. At each branch, the number indicates a bootstrap value. Black circle and Triangular index indicate the H5N6 HPAI isolates in Korea and H5N6 HPAI virus in Greece, respectively. The scale bar indicates nucleotide substitutions per site.

**References**

1. Munch M, Nielsen LP, Handberg, KJ, Jørgensen PH Detection and subtyping (H5 and H7) of avian type A influenza virus by reverse transcription-PCR and PCR-ELISA, Arch Virol. 2001: 146(1); 87-97.
2. Fereidouni SR, Starick E, Grund C, Globig A, Mettenleiter TC, Beer M, et al. Rapid molecular subtyping by reverse transcription polymerase chain reaction of the neuraminidase gene of avian influenza A viruses. Vet Microbiol. 2009: 135(3-4); 253-60. doi: 10.1016/j.vetmic.2008.09.077.
3. Hoffmann E, Stech J, Guan T, Webster RG, Perez DR. Universal primer set for the full-length amplification of all influenza A viruses. Arch Virol. 2001: 146(2); 2275-89.
4. Katoh K, Standley DM. MAFFT multiple sequence alignment software version 7: improvements in performance and usability. Mol Biol Evol, 2013: 30(4); 772-80. doi: 10.1093/molbev/mst010.
5. Nguyen LT, Schmidt HA, von Haeseler A, Minh BQ, IQ-TREE: A fast and effective stochastic algorithm for estimating maximum likelihood phylogenies. Mol Biol Evol, 2015: 32(1); 268-74. doi: 10.1093/molbev/msu300.
6. Kalyaanamoorthy S, Minh BQ, Wong TKF, von Haeseler A, Jermiin LS, ModelFinder: Fast model selection for accurate phylogenetic estimates. Nat. Methods, 2017: 14(6); 587-9. doi: 10.1083/nmeth.4285
