# Supplementary figures and images for: Characterization of a novel reassortant H5N6 highly pathogenic avian influenza virus clade 2.3.4.4 in Korea, 2017
Source: Emerg Microbes Infect. 2018 Jun 13;7:103. doi: 10.1038/s41426-018-0104-3 (PMC5997646; doi:10.1038/s41426-018-0104-3)

## Slide 1
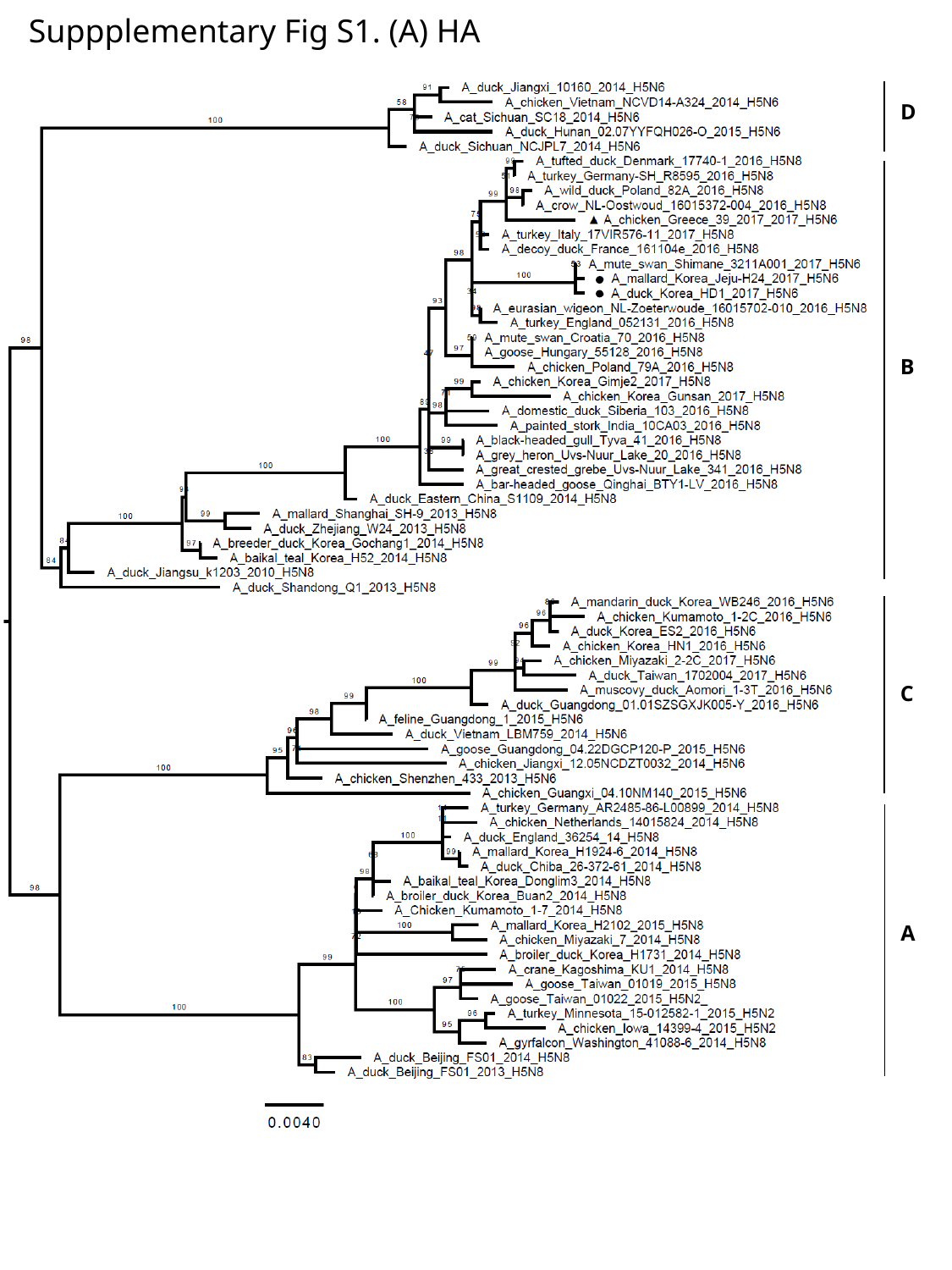

Suppplementary Fig S1. (A) HA
D
B
C
A
| |
| --- |
| |
| --- |
| |
| --- |
| |
| --- |

## Slide 2
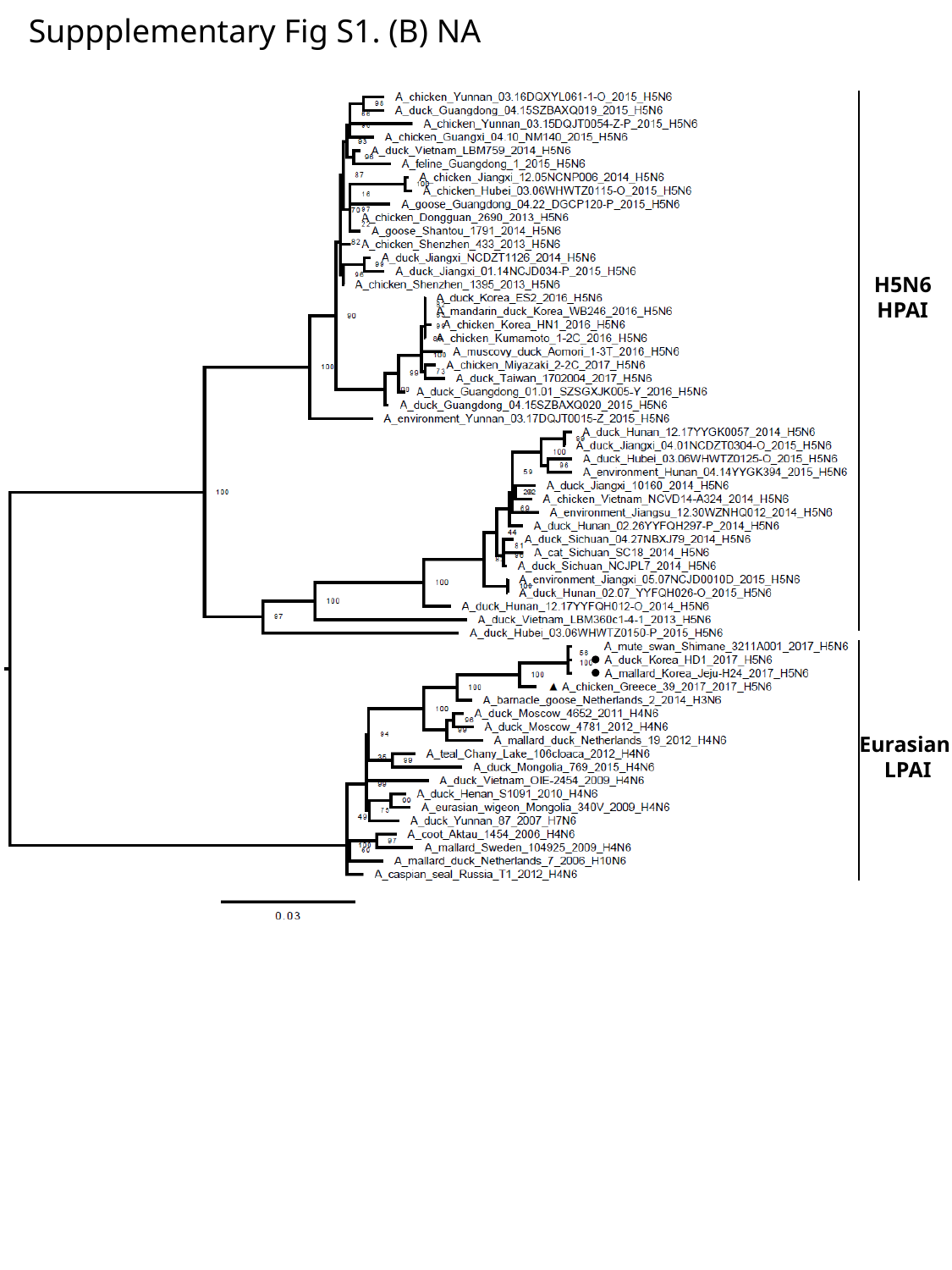

Suppplementary Fig S1. (B) NA
H5N6
HPAI
Eurasian
LPAI
| |
| --- |
| |
| --- |

Supplement: Supplementary file 2 — Supplementary Figure S1 [file 41426_2018_104_MOESM2_ESM.pptx]
