# Supplementary material for: Characterization of a novel reassortant H5N6 highly pathogenic avian influenza virus clade 2.3.4.4 in Korea, 2017
Source: Emerg Microbes Infect. 2018 Jun 13;7:103. doi: 10.1038/s41426-018-0104-3 (PMC5997646; doi:10.1038/s41426-018-0104-3)

Supplementary Fig S2. (A) PB2

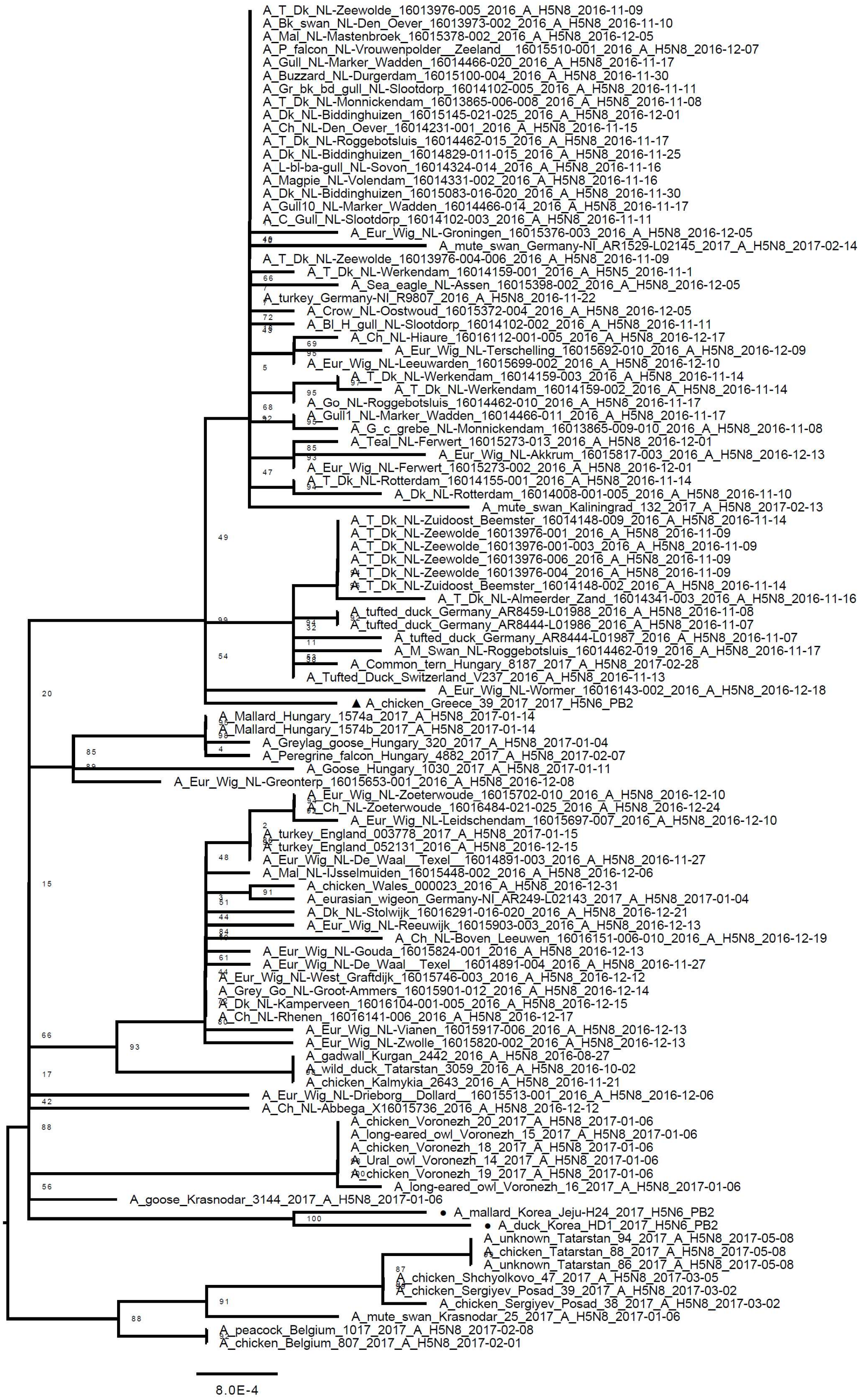

Supplementary Fig S2. (B) PB1

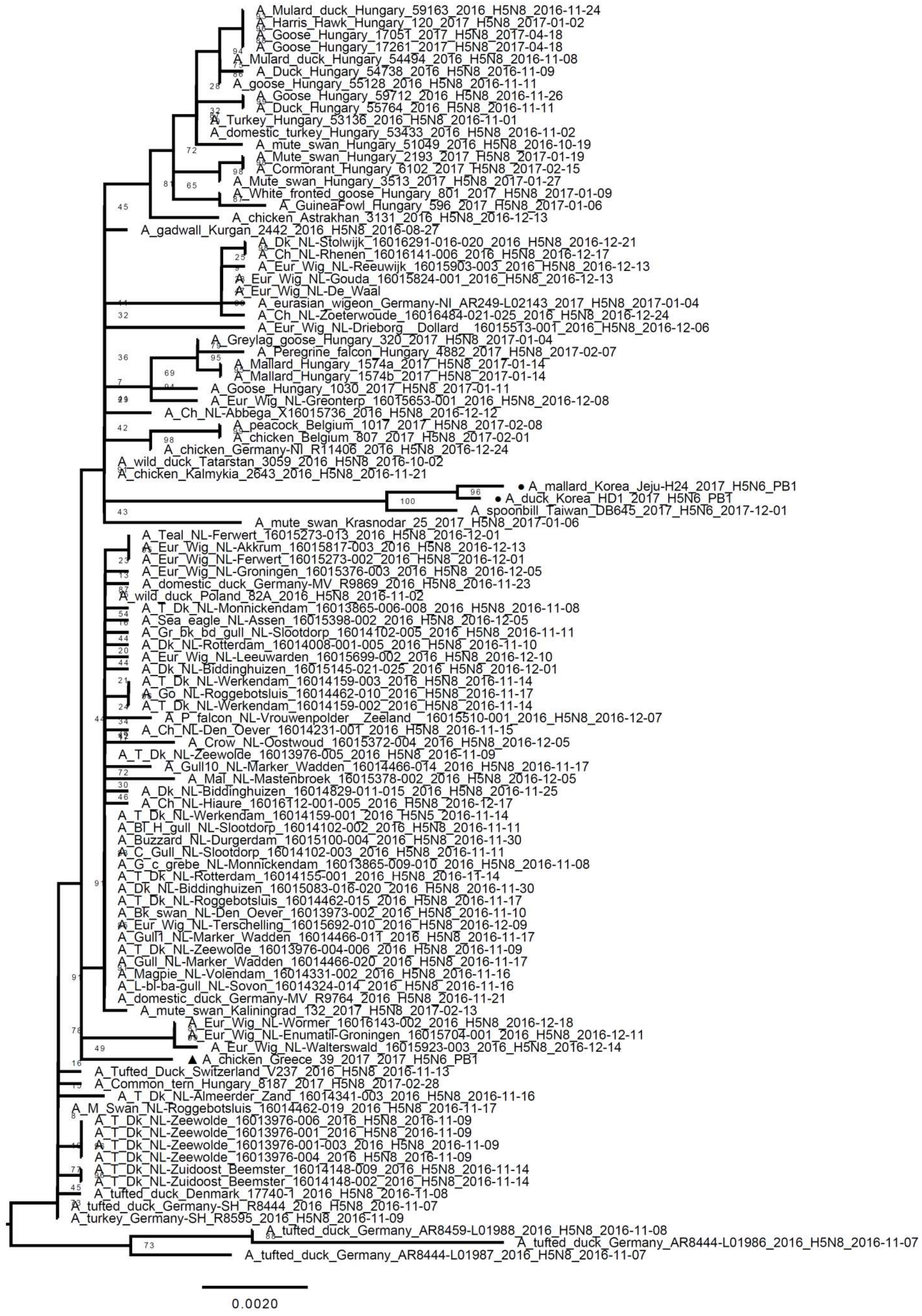

Supplementary Fig S2. (C) PA

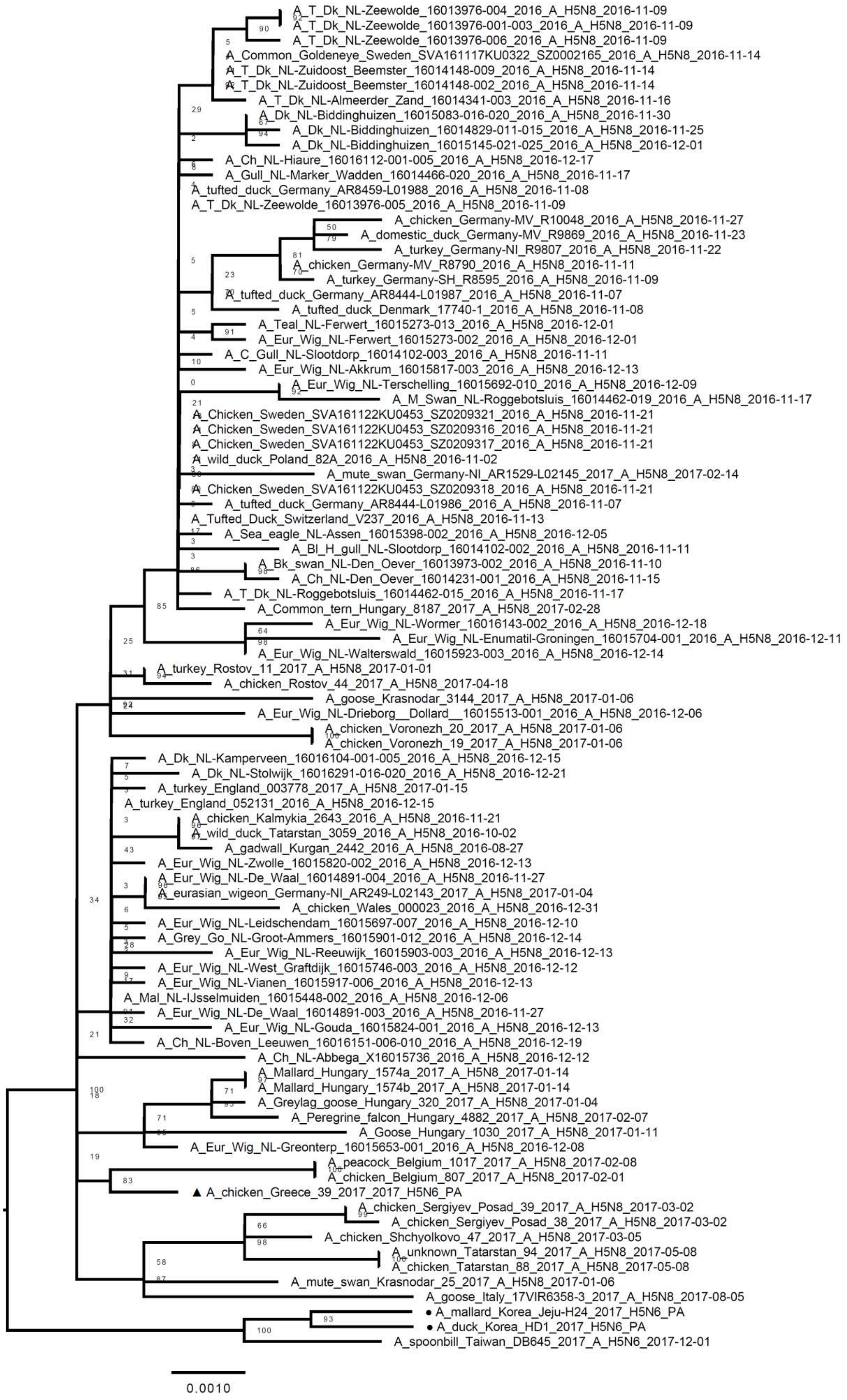

Supplementary Fig S2. (D) HA

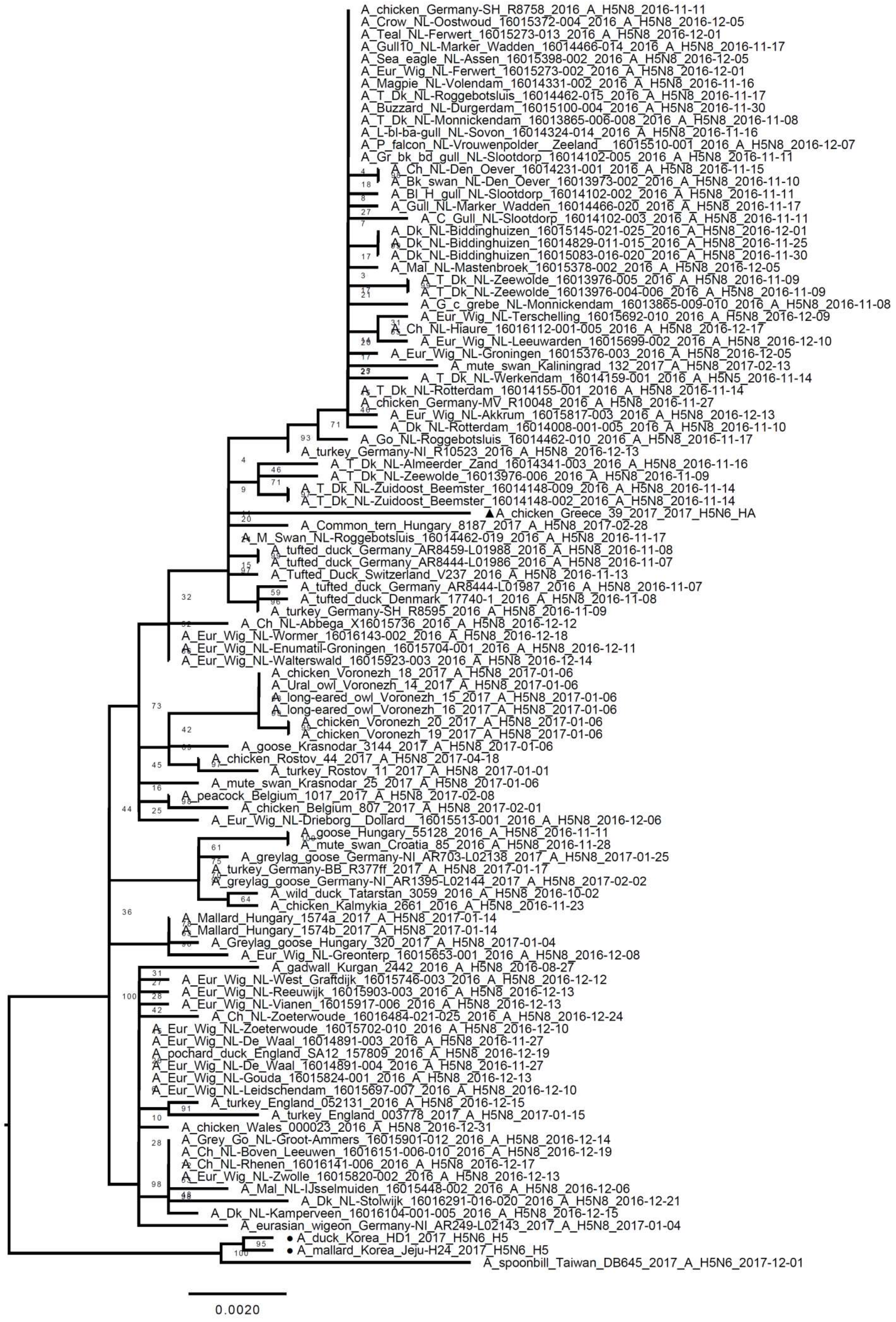

Supplementary Fig S2. (E) NP

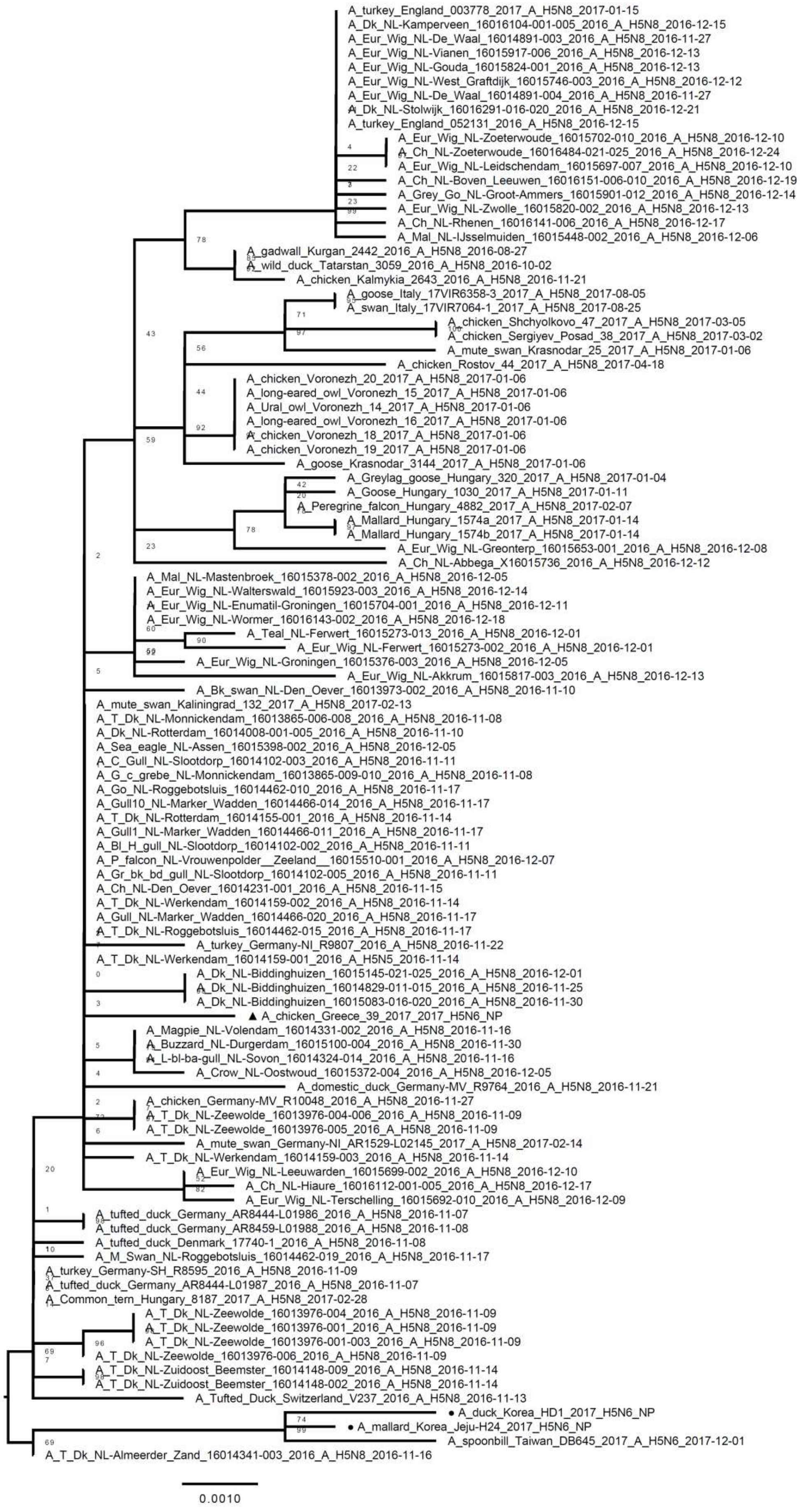

Supplementary Fig S2. (F) NA

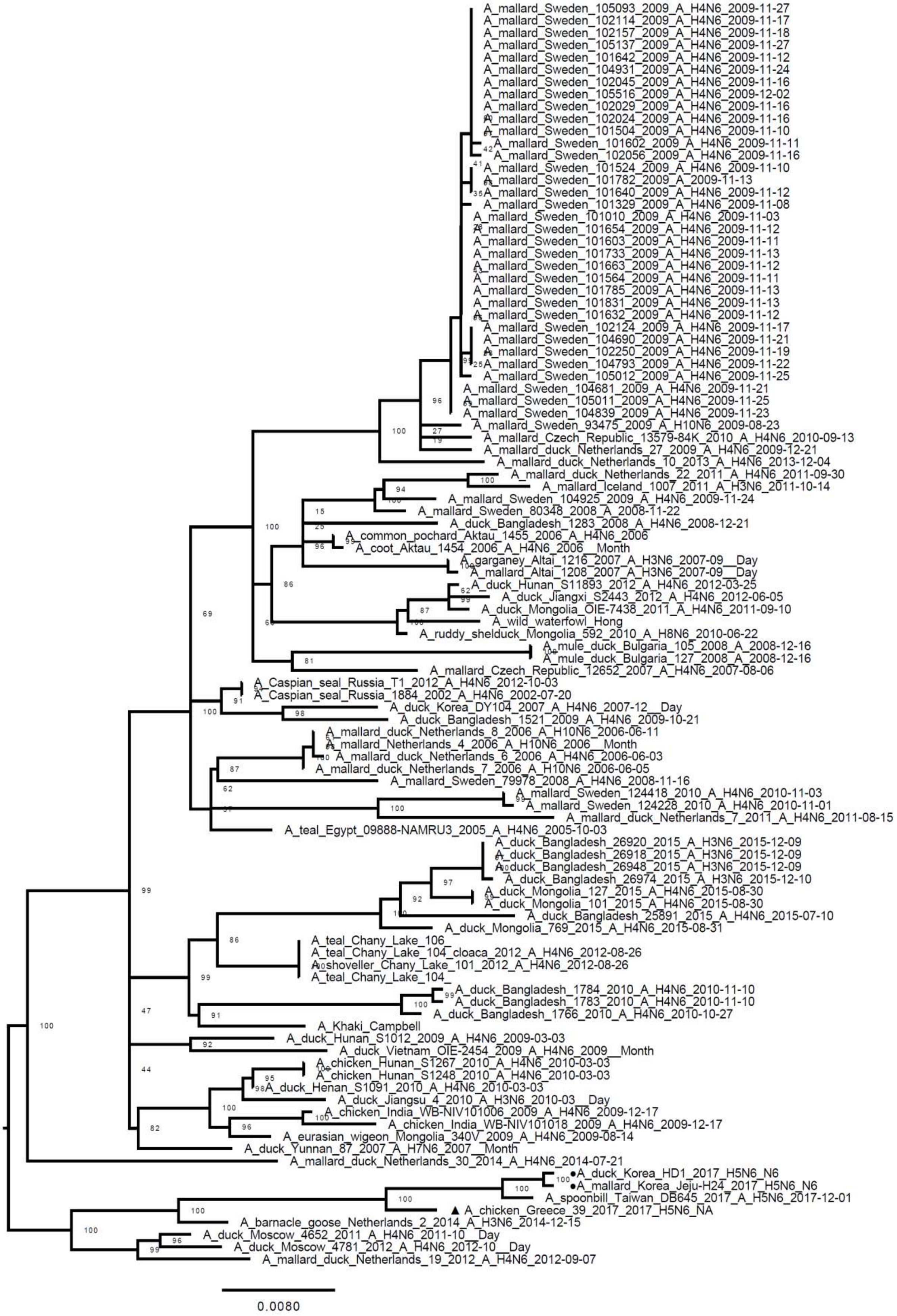

Supplementary Fig S2. (G) M

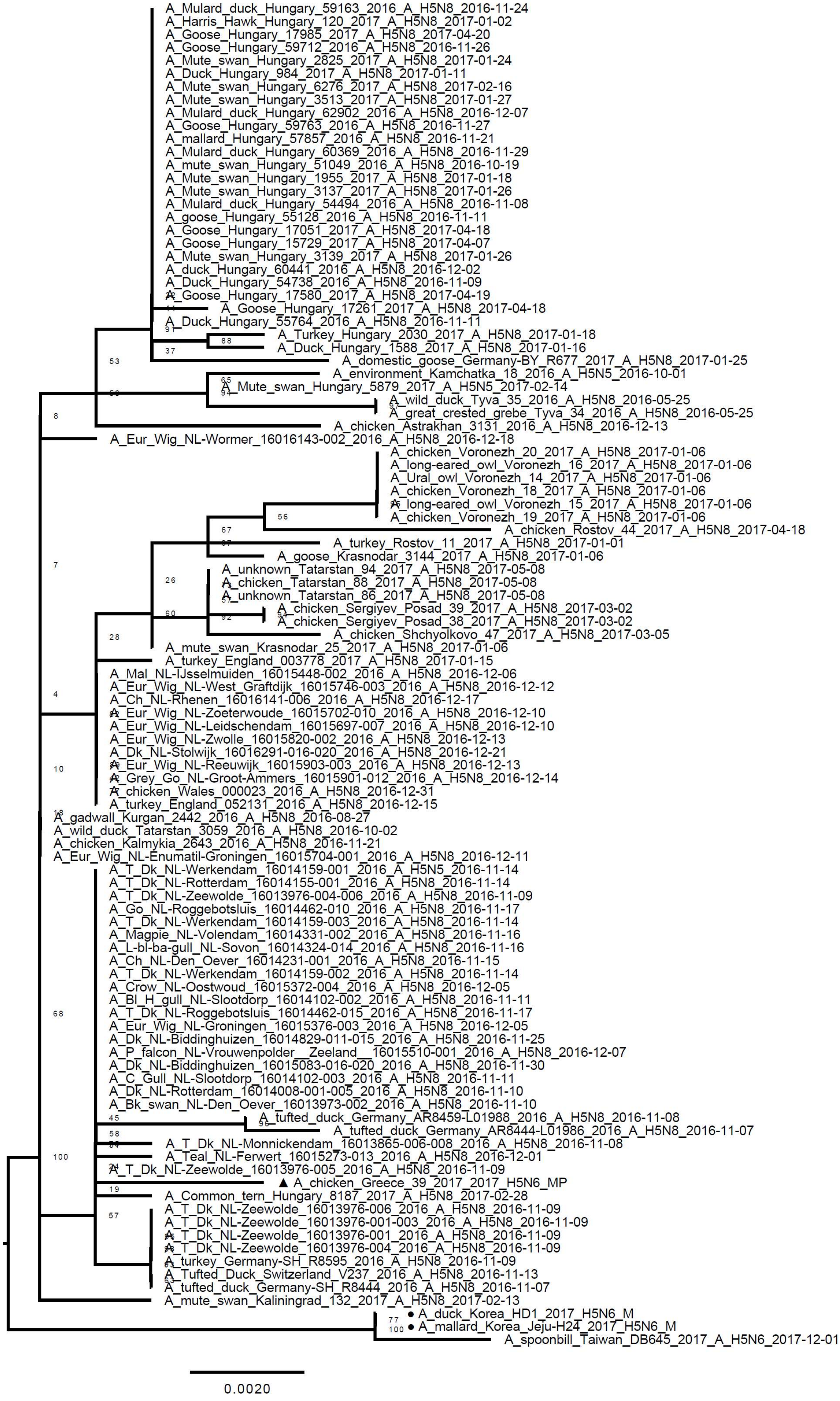

Supplementary Fig S2. (H) NS

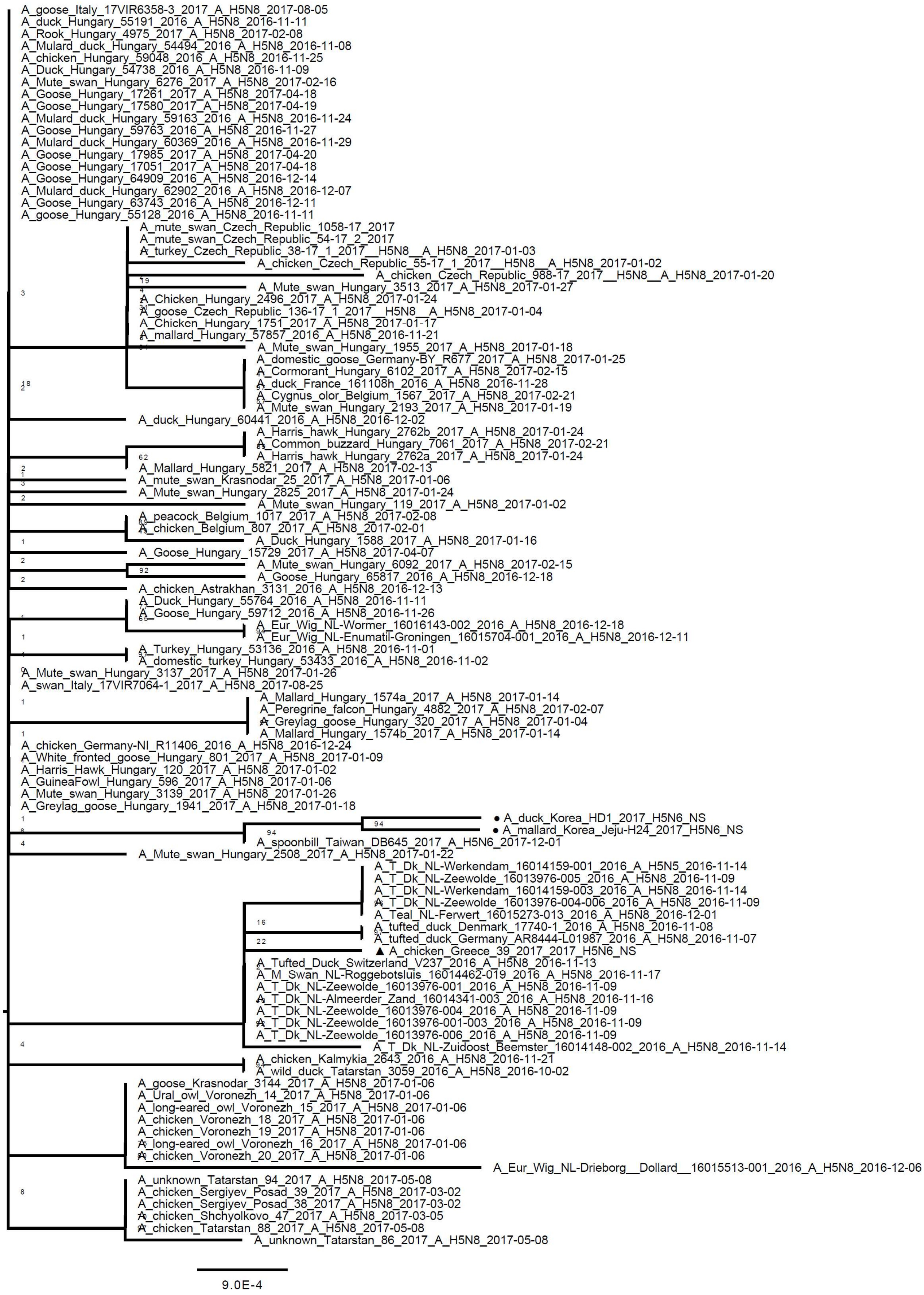

Supplement: Supplementary file 3 — Supplementary Figure S2 [file 41426_2018_104_MOESM3_ESM.pdf]
