# Supplementary material for: Characterization of a novel reassortant H5N6 highly pathogenic avian influenza virus clade 2.3.4.4 in Korea, 2017
Source: Emerg Microbes Infect. 2018 Jun 13;7:103. doi: 10.1038/s41426-018-0104-3 (PMC5997646; doi:10.1038/s41426-018-0104-3)
Supplement: Supplementary file 4 — Supplementary table S1 [file 41426_2018_104_MOESM4_ESM.pdf]

Supplementary table S1. (A) HA sequence information of H5 viruses for phylogenetic analysis.

Data source	Accession No.	Taxname
GISAID*	EPI866093	A/duck/Korea/ES2/2016(H5N6)
GISAID	EPI866132	A/mandarin_duck/Korea/WB246/2016(H5N6)
GISAID	EPI866085	A/chicken/Korea/HN1/2016(H5N6)
GISAID	EPI509698	A/breeder_duck/Korea/Gochang1/2014_H5N8
GISAID	EPI509704	A/broiler_duck/Korea/Buan2/2014_H5N8
GISAID	EPI509709	A/baikal_teal/Korea/Donglim3/2014_H5N8
GISAID	EPI573221	A/broiler_duck/Korea/H1731/2014_H5N8
GISAID	EPI573239	A/mallard/Korea/H1924-6/2014_H5N8
GISAID	EPI837542	A/mallard/Korea/H2102/2015_H5N8
GISAID	EPI760026	A/duck/Guangdong/01.01_SZSGXJK005-Y/2016_H5N6
GISAID	EPI660303	A/goose/Guangdong/04.22_DGCP120-P/2015(H5N6)
GISAID	EPI657578	A/chicken/Jiangxi/12.05_NCDZT0032/2014(H5N6)
GISAID	EPI599353	A/duck/Jiangxi/10160/2014_H5N6
GISAID	EPI661903	A/chicken/Guangxi/04.10_NM140/2015(H5N6)
GISAID	EPI599285	A/chicken/Shenzhen/433/2013_H5N6
GISAID	EPI590891	A/duck/Sichuan/NCJPL7/2014_H5N6
GISAID	EPI658951	A/duck/Hunan/02.07_YYFQH026-O/2015(H5N6)
GISAID	EPI585724	A/cat/Sichuan/SC18/2014_H5N6
GISAID	EPI517161	A/Chicken/Kumamoto/1-7/2014_H5N8
GISAID	EPI548493	A/duck/Chiba/26-372-61/2014_H5N8
GISAID	EPI553343	A/chicken/Miyazaki/7/2014_H5N8
GISAID	EPI1028076	A/mallard/Shanghai/SH-9/2013_H5N8
GISAID	EPI530985	A/duck/Zhejiang/W24/2013_H5N8
GISAID	EPI553208	A/crane/Kagoshima/KU1/2014_H5N8
GISAID	EPI891535	A/chicken/Miyazaki/2-2C/2017_H5N6
GISAID	EPI867089	A/muscovy_duck/Aomori/1-3T/2016_H5N6
GISAID	EPI881069	A/chicken/Kumamoto/1-2C/2016_H5N6
GISAID	EPI543002	A/duck/Beijing/FS01/2014_H5N8
GISAID	EPI703608	A/duck/Eastern_China/S1109/2014_H5N8
GISAID	EPI442017	A/duck/Jiangsu/k1203/2010_H5N8
GISAID	EPI596616	A/duck/Vietnam/LBM759/2014_H5N6
GISAID	EPI750122	A/goose/Taiwan/01019/2015_H5N8
GISAID	EPI915870	A/duck/Taiwan/1702004/2017_H5N6
GISAID	EPI750154	A/goose/Taiwan/01022/2015_H5N2
GISAID	EPI926613	A/domestic_duck/Siberia/103/2016_H5N8
GISAID	EPI773757	A/great_crested_grebe/Uvs-Nuur_Lake/341/2016_H5N8
GISAID	EPI823756	A/black-headed_gull/Tyva/41/2016_H5N8
GISAID	EPI836606	A/grey_heron_/Uvs-Nuur_Lake/20/2016_H5N8
GISAID	EPI858844	A/painted_stork/India/10CA03/2016_H5N8
GISAID	EPI869927	A/chicken/Poland/79A/2016_H5N8
GISAID	EPI861568	A/mute_swan/Croatia/70/2016_H5N8
GISAID	EPI547673	A/duck/England/36254/14_H5N8
GISAID	EPI909364	A/tufted_duck/Denmark/17740-1/2016_H5N8
GISAID	EPI860231	A/wild_duck/Poland/82A/2016_H5N8
GISAID	EPI860519	A/goose/Hungary/55128/2016_H5N8
GISAID	EPI774121	A/Bar-headed_Goose/Qinghai/BTY1-LV/2016_H5N8
GISAID	EPI954575	A/turkey/Italy/17VIR576-11/2017_H5N8
GISAID	EPI1019638	A/Eur_Wig/NL-Zoeterwoude/16015702-010/2016_H5N8
GISAID	EPI869687	A/decoy_duck/France/161104e/2016_H5N8
GISAID	EPI1019446	A/Crow/NL-Oostwoud/16015372-004/2016_H5N8
GISAID	EPI861011	A/turkey/Germany-SH/R8595/2016_H5N8
GISAID	EPI552746	A/turkey/Germany/AR2485-86-L00899/2014_H5N8
GISAID	EPI544892	A/duck/Shandong/Q1/2013_H5N8
GISAID	EPI573171	A/Chicken/Netherlands/14015824/2014_H5N8
GISAID	EPI569390	A/gyrfalcon/Washington/41088-6/2014_H5N8
GISAID	EPI778498	A/turkey/Minnesota/15-012582-1/2015_H5N2
GISAID	EPI542617	A/duck/Beijing/FS01/2013_H5N8
GISAID	EPI760089	A/feline/Guangdong/1/2015_H5N6
GISAID	EPI868848	A/turkey/England/052131/2016_H5N8
GISAID	EPI620450	A/chicken/Iowa/14399-4/2015_H5N2
GISAID	EPI894943	A/chicken/Vietnam/NCVD14-A324/2014(H5N6)
Genbank	LC335983	A/mute swan/Shimane/3211A001/2017(H5N6)
GISAID	EPI1123351	A/chicken/Korea/Gimje2/2017(H5N8)
GISAID	EPI1123343	A/chicken/Korea/Gunsan/2017(H5N8)
GISAID	EPI1123317	A/duck/Korea/HD1/2017(H5N6)
GISAID	EPI1123335	A/mallard/Korea/Jeju-H24/2017(H5N6)
Genbank	KJ508961	A/baikal teal/Korea/H52/2014(H5N8)

* We acknowledge the authors, originating and submitting laboratories of the sequences from GISAID's EpiFlu™ Database on which this research is based. Contact details of submitters can be found at: http://platform.giscaid.org/epi3/frontend#326742.

Supplementary table S1. (B) NA sequence information of HxN6 viruses for phylogenetic analysis.

Data source	Accession No.	Taxname
Genbank	KP286103	A/chicken/Dongguan/2690/2013(H5N6)
Genbank	KP284975	A/chicken/Shenzhen/1395/2013(H5N6)
Genbank	KP285327	A/goose/Shantou/1791/2014(H5N6)
GISAID*	EPI596618	A/duck/Vietnam/LBM759/2014(H5N6)
GISAID	EPI664220	A/duck/Jiangxi/01.14NCJD034-P/2015(H5N6)
Genbank	KP090449	A/duck/Jiangxi/NCDZT1126/2014(H5N6)
GISAID	EPI664580	A/chicken/Yunnan/03.16DQXYL061-1-O/2015(H5N6)
GISAID	EPI661902	A/chicken/Guangxi/04.10NM140/2015(H5N6)
GISAID	EPI664388	A/duck/Guangdong/04.15SZBAXQ019/2015(H5N6)
GISAID	EPI658494	A/chicken/Jiangxi/12.05NCNP006/2014(H5N6)
GISAID	EPI658502	A/chicken/Hubei/03.06WHWTZ0115-O/2015(H5N6)
GISAID	EPI659094	A/chicken/Yunnan/03.15DQJT0054-Z-P/2015(H5N6)
GISAID	EPI866095	A/duck/Korea/ES2/2016(H5N6)
GISAID	EPI866134	A/mandarin duck/Korea/WB246/2016(H5N6)
GISAID	EPI866087	A/chicken/Korea/HN1/2016(H5N6)
GISAID	EPI661958	A/duck/Guangdong/04.15SZBAXQ020/2015(H5N6)
GISAID	EPI664572	A/environment/Yunnan/03.17DQJT0015-Z/2015(H5N6)
GISAID	EPI657017	A/duck/Hunan/02.26YYFQH297-P/2014(H5N6)
GISAID	EPI657377	A/duck/Hunan/12.17YYGK0057/2014(H5N6)
GISAID	EPI664492	A/duck/Jiangxi/04.01NCDZT0304-O/2015(H5N6)
GISAID	EPI664380	A/environment/Jiangsu/12.30WZNHQ012/2014(H5N6)
GISAID	EPI658654	A/duck/Hubei/03.06WHWTZ0125-O/2015(H5N6)
GISAID	EPI658654	A/duck/Sichuan/04.27NBXJ79/2014(H5N6)
GISAID	EPI664588	A/environment/Hunan/04.14YYGK394/2015(H5N6)
GISAID	EPI599355	A/duck/Jiangxi/10160/2014(H5N6)
GISAID	EPI658038	A/environment/Jiangxi/05.07NCJD0010D/2015(H5N6)
GISAID	EPI656537	A/duck/Hunan/12.17YYFQH012-O/2014(H5N6)
Genbank	LC010698	A/duck/Vietnam/LBM360c1-4-1/2013(H5N6)
GISAID	EPI664532	A/duck/Hubei/03.06WHWTZ0150-P/2015(H5N6)
Genbank	MF146256	A/barnacle goose/Netherlands/2/2014(H3N6)
Genbank	KX518716	A/duck/Moscow/4652/2011(H4N6)
Genbank	KX530515	A/duck/Moscow/4781/2012(H4N6)
Genbank	KX978671	A/mallard duck/Netherlands/19/2012(H4N6)
Genbank	KJ847705	A/caspian seal/Russia/T1/2012(H4N6)
Genbank	KF259617	A/duck/Yunnan/87/2007(H7N6)
Genbank	MF147888	A/mallard duck/Netherlands/7/2006(H10N6)
Genbank	KU160941	A/duck/Henan/S1091/2010(H4N6)
Genbank	FJ434371	A/coot/Aktau/1454/2006(H4N6)
Genbank	KF667698	A/eurasian wigeon/Mongolia/340V/2009(H4N6)
Genbank	CY166034	A/mallard/Sweden/104925/2009(H4N6)
GISAID	EPI760028	A/duck/Guangdong/01.01 SZSGXJK005-Y/2016(H5N6)
GISAID	EPI660302	A/goose/Guangdong/04.22 DGCP120-P/2015(H5N6)
GISAID	EPI599287	A/chicken/Shenzhen/433/2013(H5N6)
GISAID	EPI590864	A/duck/Sichuan/NCJPL7/2014(H5N6)
GISAID	EPI658950	A/duck/Hunan/02.07 YYFQH026-O/2015(H5N6)
GISAID	EPI585726	A/cat/Sichuan/SC18/2014(H5N6)
GISAID	EPI891534	A/chicken/Miyazaki/2-2C/2017(H5N6)
GISAID	EPI867088	A/muscovy duck/Aomori/1-3T/2016(H5N6)
GISAID	EPI881068	A/chicken/Kumamoto/1-2C/2016(H5N6)
GISAID	EPI915872	A/duck/Taiwan/1702004/2017(H5N6)
GISAID	EPI760091	A/feline/Guangdong/1/2015(H5N6)
Genbank	LC335985	A/mute swan/Shimane/3211A001/2017(H5N6)
GISAID	EPI823917	A/teal/Chany_Lake/106cloaca/2012(H4N6)
Genbank	AB545604	A/duck/Vietnam/OIE-2454/2009(H4N6)
Genbank	LC121454	A/duck/Mongolia/769/2015(H4N6)
GISAID	EPI1123319	A/duck/Korea/HD1/2017(H5N6)
GISAID	EPI1123337	A/mallard/Korea/Jeju-H24/2017(H5N6)
GISAID	EPI894945	A/chicken/Vietnam/NCVD14-A324/2014(H5N6)

* We acknowledge the authors, originating and submitting laboratories of the sequences from GISAID's EpiFlu™ Database on which this research is based. Contact details of submitters can be found at: http://platform.giscaid.org/epi3/frontend#326742.
